# Membrane fractionation and characterization of shiitake (*Lentinula edodes*) polysaccharides with in vitro antifungal activity against phytopathogens

**DOI:** 10.1007/s11356-026-38143-7

**Published:** 2026-08-19

**Authors:** Barbara Vargas da Rosa, Talize Helena Bresolin, Jonathan Barcelos de Freitas, Raquel Cristine Kuhn

**Affiliations:** Department of Chemical Engineering, Federal University of Santa Maria, 1000 Roraima Avenue, Santa Maria, 97105-900 Brazil

**Keywords:** *Lentinula edodes*, Polysaccharides, Membrane ultrafiltration, Antifungal activity, Fungal phytopathogens, Sustainable agriculture

## Abstract

The shiitake mushroom (*Lentinula edodes*) is a rich source of bioactive polysaccharides with potential applications in sustainable agriculture, particularly as environmentally friendly alternatives to synthetic fungicides. This study aimed to employ membrane separation technology to streamline the recovery of polysaccharides from shiitake mushrooms and evaluate their antifungal activity against phytopathogens. Crude aqueous extracts and membrane-fractionated polysaccharides were tested in vitro against *Colletotrichum gloeosporioides*, *Fusarium oxysporum*, *Macrophomina phaseolina*, *Rhizoctonia solani*, and *Sclerotinia sclerotiorum*. The crude extract exhibited the strongest inhibition, with 13.0 ± 0.07% and 5.87 ± 0.03% growth reduction against *R. solani* and *M. phaseolina*, respectively. Membrane ultrafiltration successfully concentrated polysaccharides within a targeted molecular weight range, with glucose, rhamnose, and arabinose confirmed as dominant monosaccharides via HPLC. However, fractionation slightly reduced bioactivity compared to the crude extract. These findings demonstrate that shiitake-derived polysaccharides exhibit measurable in vitro antifungal activity and highlight their potential for further investigation as bioactive natural compounds. Furthermore, the results support the use of membrane separation technology as a sustainable strategy for the recovery and concentration of bioactive mushroom-derived polysaccharides.

## Introduction

Mushroom polysaccharides, present as a structural component of the cell wall (Xu et al. 2014), are among the most significant bioactive components found in fungi (Ali et al. 2024). With the growing popularity of healthy diets, several bioactive components of fungi have attracted increasing attention in recent years, such as polysaccharides (Ali et al. 2024). In 2022, approximately 48.3 million tons of mushrooms was produced globally, with an annual increase of about 8–9% over the past decades (Araújo-Rodrigues et al. 2024). *Lentinula edodes* (shiitake) accounts for 17% of the global supply of edible fungi and it is currently the second most popular edible mushroom in the global market due to its taste, nutritional benefits, and therapeutic value (Sheng et al. 2021).

The popularity of shiitake is attributed to the presence of molecules with a wide variety of health benefits, being considered a functional food (Bisen et al. 2010). Several studies report the presence of compounds with bioactive properties of interest to the food, pharmaceutical, and medical industries (Sheng et al. 2021), including antioxidant (da Rosa et al. 2024; Ren et al. 2018), antifungal (Yehia 2022), anticancer (Li et al. 2018; Yehia 2022), antidiabetic (Yehia 2022), anti-inflammatory (Ren et al. 2018; Zhang et al. 2023), anti-aging (Wang et al. 2015), antiviral (Zhao et al. 2018), and immunomodulatory activities (Li et al. 2018). Among these properties, the antifungal activity is, so far, one of the least explored for this mushroom; according to Yehia (2022), as shiitake polysaccharides are natural and nontoxic to humans, more studies should be conducted to explore a practical application in the prevention and protection of fungal infections of plants, humans, and animals (Yehia 2022).

On the other hand, one of the main challenges in agriculture is the damage caused by pests, including insects, bacteria, and fungi. Among these, fungi are the main pathogens of crops used for food and feed production (Manzanares et al. 2024), causing significant economic and production losses and impacting food quality and safety (Montesinos and Bardají 2008; Khan et al. 2020). Chemical control is the main method used to combat these pathogens, but its overuse results in chemical pollution, risks to the environment, human health, and animals (Brun et al. 2022). Furthermore, several phytopathogenic microorganisms have developed resistance to these synthetic chemicals, rendering them more difficult to control (De la Cruz Quiroz et al. 2015). Therefore, sustainable agricultural practices that ensure food security and preserve ecosystem services are essential to reduce dependence on synthetic fungicides (Rehman et al. 2022). In this context, naturally derived bioactive compounds have attracted increasing attention as potential alternatives or complementary tools for reducing reliance on conventional synthetic fungicides (Assefa et al. 2026).

Thereby, the present study aimed to obtain polysaccharides from expired shiitake mushrooms using a membrane separation process. Additionally, the use of expired shiitake mushrooms contributes to the valorization of biological materials that would otherwise have limited commercial value, aligning with current efforts toward more sustainable and resource-efficient utilization of biomass. Ultrafiltration membranes with different molecular weight cutoffs were employed to evaluate the concentration and fractionation of these compounds. The fractions were subsequently characterized and evaluated for their in vitro antifungal activity against phytopathogenic fungi. In addition, the study investigated the applicability of membrane separation as a sustainable approach for the recovery and concentration of bioactive polysaccharides from shiitake mushrooms while providing insights into the properties of fractions and preliminary information for future investigation into their potential agricultural applications.

## Materials and methods

### Shiitake mushrooms obtention and preparation

The shiitake mushrooms were obtained from a local distributor, aged within 2 to 3 weeks of their expiration date. After receiving the raw material, the mushrooms were dried in an oven at 50 °C, milled to a Tyler 10 (1.7 mm) particle size, and kept at 4 °C until further extraction.

### Polysaccharides extraction

The extraction was performed according to Vezaro et al. (2022). A high-intensity ultrasound processor (Model UP 400S; Hielscher Ultrasonics, Teltow, Germany) was used; the extraction was carried out using 2.5 g of sample with 100 ml of distilled water at 25 °C for 1 h. The ultrasound-assisted extraction was performed at an amplitude of 100% and pulse cycle of 0.75. Afterwards, the solution was filtered using Whatman filter paper, the cells were discarded, and the extract was stored in the refrigerator at −20 °C until use.

### Membrane separation process

This process was performed in batch mode and consists of the complete feed flow being forced through the membrane, with the filtered material remaining accumulated on the membrane surface. Fifty milliliters of shiitake extract was used for each assay, to avoid clogging due to the accumulated material and to avoid interference with the filtration process. The equipment used was a dead-end stirred cell filtration connected to an ultrathermostatic bath maintained at 25 °C for temperature control. Industrial nitrogen was used to apply pressure as the driving force to enable transport through the membrane in the system, which was controlled by a valve and kept constant throughout the process. A magnetic agitator was used to avoid the increase of polarization layer and fouling on the membrane surface, which increases resistance to permeation (Xu et al. 2014). The polymeric membranes used in this experiment were the ultrafiltration membranes with nominal molecular weight cutoff (MWCO) of 150 kDa (UP150 P, PES, Microdyn Nadir, Germany), 20 kDa (UP020 P, PES, Microdyn Nadir, Germany), and 5 kDa (UP005 P, PES, Microdyn Nadir, Germany).

### In vitro antifungal activity against phytopathogens

The bioactive polysaccharides present in the shiitake extract were evaluated for their ability to inhibit the growth of fungi that are challenging to eradicate and primarily attack soybeans, but are also present in various crops. The tests were conducted in vitro using the fungi *Colletotrichum gloeosporioides*, *Fusarium oxysporum*, *Macrophomina phaseolina*, *Rhizoctonia solani*, and *Sclerotinia sclerotiorum*, provided by the Department of Phytosanitary Defense of Federal University of Santa Maria (UFSM, Santa Maria, Brazil).

**Fig. 1:**
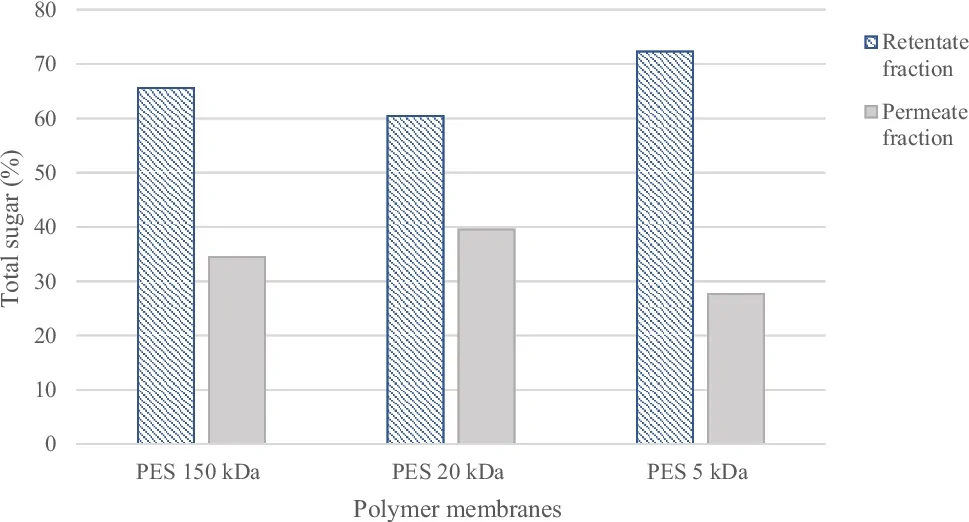
Total sugar of the retentate and permeate membrane fractions

The fungi were stored in potato dextrose agar (PDA) culture medium, and the technique used was agar diffusion method according to Balouiri et al. (2016). Initially, the samples were filtered in a membrane (0.22 µm) for cell removal. For the antifungal assays, 100 µl of each undiluted sample was applied onto solidified PDA plates and spread with a sterile cell spreader until dry. The crude extract used in the assay contained 2578.21 ± 0.05 µg/ml of total sugars. The total sugar concentrations of the membrane fractions used in the assays are presented in Fig. 1. Subsequently, inoculation was carried out by transferring a 6-mm-diameter mycelial disc from a pure culture of the evaluated microorganism.

The plates were left in a BOD incubator with light at 25 °C. Blanks only with PDA medium were performed for comparison. After further incubation, the diameters of fungal growth in control and sample plates were measured, and the antifungal effect was estimated by Eq. 1.

1 $$\mathrm{A}\mathrm{n}\mathrm{t}\mathrm{i}\mathrm{f}\mathrm{u}\mathrm{n}\mathrm{g}\mathrm{a}\mathrm{l} \,\mathrm{a}\mathrm{c}\mathrm{t}\mathrm{i}\mathrm{v}\mathrm{i}\mathrm{t}\mathrm{y} \,\left(\mathrm{\%}\right)=\frac{{D}_{B}-{D}_{A}}{{D}_{B}}*100$$

where *D*_B_ is the diameter of growth in blank plate and *D*_A_ is the diameter of growth in the plate containing tested antifungal agent.

The fungi with highest inhibitory effects were chosen to be evaluated in the permeate and retentate fractions obtained from the separation of compounds by membrane separation process.

### Total sugar determination

The phenol-sulfuric acid method proposed by DuBois et al. (1956) was used to evaluate the polysaccharide content. One thousand microliters of the sample was added in a test tube; then, 25 µl of 80% phenol and 2500 µl of sulfuric acid were added; after stirring in a vortex, the samples were taken to a bath at 25 °C for 15 min (DuBois et al. 1956). Glucose was used as standard and the absorbance of samples was read in a UV-Vis spectrophotometer (UV-2600 Shimadzu) with a wavelength of 485 nm.

### Fourier transform infrared spectroscopy (FTIR) analysis

Fourier-transform infrared spectroscopy analysis was used to characterize the lyophilized samples to evidence the presence of functional groups. The IR spectroscopy was performed using an IR Prestige-21 apparatus (Shimadzu) operating in the wavenumber range from 4500 to 400 cm^−1^ and sample prepared using the KBr pellets technique (in the form of discs). The resolution and the number of scans of the FTIR spectrum are 2.0 and 45, respectively.

### Determination of molecular weight

The molecular weight of the polysaccharides in the permeated and retentate fractions of the shiitake extract was evaluated. The analyses were performed using a high-performance liquid chromatography (HPLC) equipped with Polysep-GFC-P 4000 column and a Polysep-GFC-P 1000 guard column (7.8 × 300 mm, Phenomenex), with a refractive index detector (RID 10 A, Shimadzu). Dextran analytical standards were used to obtain the standard curve according to the methodology of Lin and Tsai (2019). Pure water was used as the mobile phase at a flow rate of 1.0 ml/min, with a 20 µl of sample injection volume, while the system was maintained at a temperature of 30 °C.

### Monosaccharide analysis

Analysis of the monosaccharide composition was performed for a better understanding of the structure of the polysaccharides separated by membrane separation process. Polysaccharide samples were hydrolyzed at 100 °C using 2 M hydrochloric acid and after an ice bath neutralized with 2 M sodium hydroxide. After hydrolysis, the solutions were filtered in 0.22 µm polytetrafluoroethylene (PTFE) membrane for the chromatographic analysis. The technique used to determine monosaccharide composition by HPLC was according to the one described by Abaide et al. (2019) with some modifications. The samples were injected into the HPLC (Proeminence UFLCXR, Shimadzu) equipped with a refractive index detector (RID 10 A, Shimadzu) and Aminex HPX-87H column (300 × 7.8 mm, Bio-Rad). The mobile phase (0.005 M sulfuric acid solution) was isocratically eluted at 0.6 ml/min; the column temperature was maintained at 65 °C. Monosaccharide quantifications were carried out through the peak area using response factors obtained with standard neutral monosaccharides (D-galactose, D-mannose, L-arabinose, D-fructose, D-glucose, D-xylose, L-rhamnose).

### Statistical analysis

Data were expressed as arithmetic mean ± standard deviation. Differences were statistically tested by Tukey HSD test (*p* < 0.05) after performing one-way analysis of variance (ANOVA) using STATISTICA 12 ® (Statsoft Inc., USA) at 95% confidence level (*p* < 0.05).

## Results and discussions

### Yield and fractionation of shiitake extraction using membrane separation process

Ultrasound-assisted extraction was applied after drying and milling in the expired shiitake mushrooms. This methodology was chosen for its extraction efficiency and its environmentally friendly characteristics. During this process, due to cavitation and energy transfer, the mushroom cells are ruptured, increasing the mass transfer of molecules to the solvent (Soria and Villamiel 2010; Chemat et al. 2017; da Rosa et al. 2021). This enables the attainment of a higher concentration of polysaccharides and facilitates the extraction of molecules that would require extended extraction times or might otherwise be inaccessible from the fungal matrix with traditional extraction. In this process, an initial concentration of 2578.21 ± 0.05 µg/mL of total sugar was obtained for the shiitake crude extract.

Ultrafiltration membranes were employed to fractionate the shiitake crude extract, aiming to increase its yield and potentially enable molecular applications. Membranes with cutoff size of 150 kDa, 20 kDa, and 5 kDa were used in fractionated assays, where permeate and retentate fractions were collected, each accounting for half of the total filtered volume. Figure 1 shows the sugar concentration in each fraction; the retentate fractions have a higher concentration of sugars. Half of the initial sample was filtered, resulting in a reduced sample volume and retention of all molecules unable to cross the membrane in the retentate fraction. The sample with highest concentration of sugars in the retentate fractions was using the 5 kDa membrane, which indicates that most of the exopolysaccharides are large enough to be unable to cross the membrane, so permeated fraction is poor in polysaccharides.

The 20-kDa permeate fraction exhibited a higher concentration of total sugars due its pore size, which facilitated molecule passage and thereby required less pressure for permeation. In contrast, the 150-kDa membrane, with its larger pore size that allows the passage of larger molecules, may have become clogged, resulting in a decrease in molecular permeation. The pressure applied in both processes was the same; however, the smaller pore size of the 20-kDa membrane likely affected the flow, resulting in the observed difference.

Other studies also used the membrane separation technique to separate shiitake compounds. Morales et al. (2019) carried out a process to obtain β-glucan-rich extracts from hot water extraction of shiitake mushroom powder. According to the authors, the extracts might be used in novel functional foods due to their high content of hypocholesterolemic compounds (Morales et al. 2019). Microfiltration membranes were used to clarify the extract, and nanofiltration was used to concentrate the polysaccharide molecules from the ethanolic precipitation of the aqueous extract of shiitake. This process did not significantly affect the total carbohydrate content, and did not assist a separation of the polysaccharides.

Tang et al. (2020) obtained three polysaccharide fractions using ultrafiltration membranes operating in crossflow filtration; however, the polysaccharide fractions still presented residues (Tang et al. 2020). Crossflow ultrafiltration was also used in the research of Sari et al. (2018) to separate fractions of the cold aqueous extract of the shiitake mushroom. This work showed evidence that certain fractions from an aqueous cold-water extract from *L. edodes* have high potential as anticancer compounds, indicating that separation may be interesting to obtain better results (Sari et al. 2018).

Ultrafiltration membranes were also used to separate proteins from shiitake extract. Schimpf and Schulz (2016) separated proteins from spent growth substrate of *Lentinula edodes* cultivation that can be used as enzymes for conversion of lignocellulosic biomass (Schimpf and Schulz 2016). Kong et al. (2019) used ultrafiltration membranes and gel filtration chromatography column to obtain umami peptides (Kong et al. 2019).

No work was achieved that obtained purified compounds using only membrane separation for shiitake extract concentration. This limitation is primarily due to the performance of the process; this phenomenon can be explained by Su et al. (2012) who demonstrated that ultrafiltration could not separate small molecules efficiently, because membranes perform poorly with compounds of similar molecular weights (Su et al. 2012; Kong et al. 2019).

In this study, the separation performed by membranes was important to identify which fractions were obtained and the composition of each fraction. Therefore, characterization analyses were performed to identify molecular weight and monosaccharide composition and Fourier transform analysis to characterize the major functional groups of the extracted polysaccharides. Subsequently, the extracts were evaluated for their in vitro antifungal activity as a preliminary assessment of their potential agricultural application and to determine whether membrane ultrafiltration improved the biological activity of the fractions.

### Antifungal activity

In vitro assays were carried out to analyze the antifungal capacity of the extract from shiitake mushroom against phytopathogen fungi, and the possible use of the extract in agricultural crops.

**Fig. 2:**
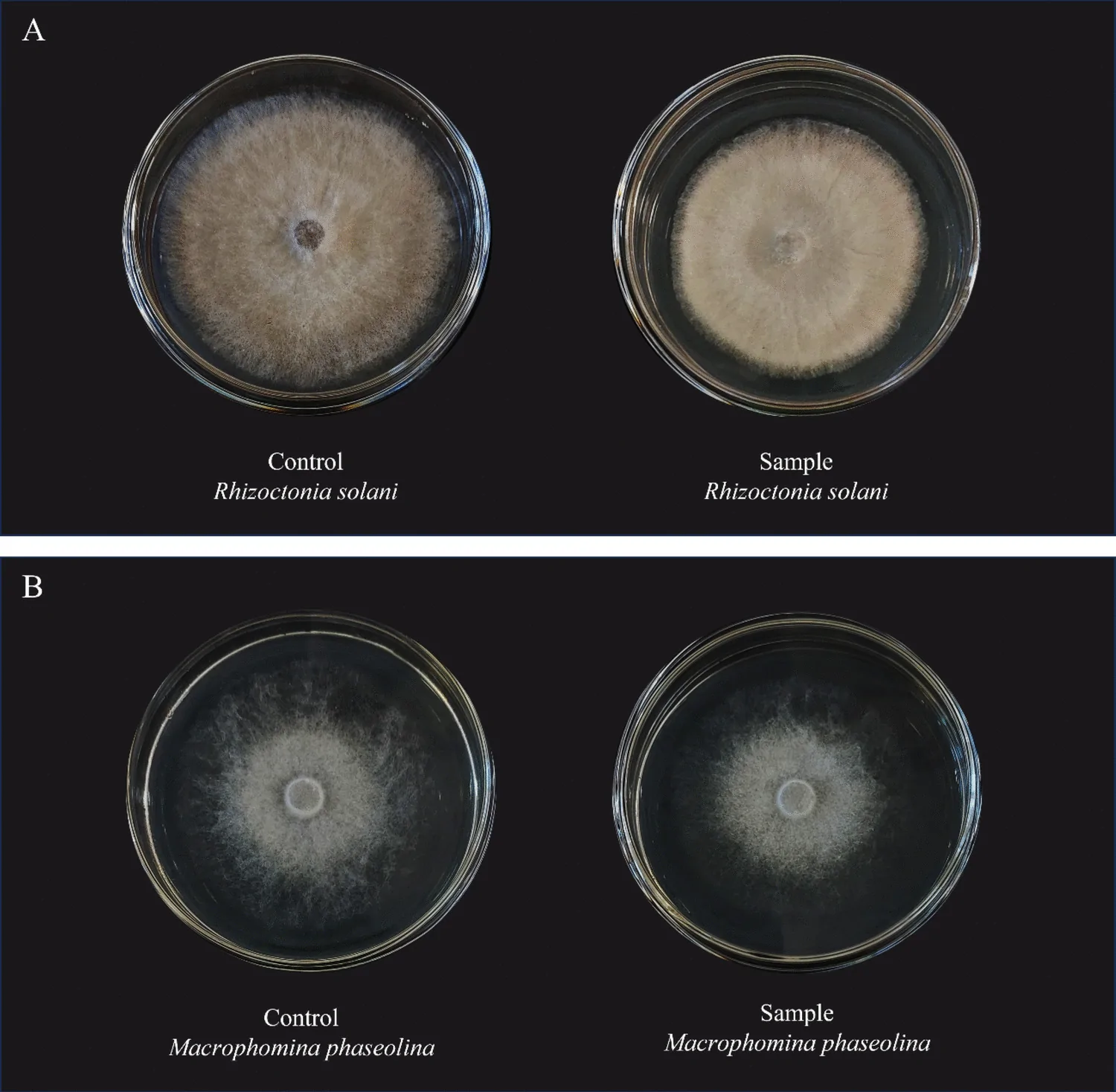
Inhibitory effects of shiitake crude extract against **A** *Rhizoctonia solani* and **B** *Macrophomina phaseolina* and control

The experiments were performed on the fungi *Rhizoctonia solani*, *Macrophomina phaseolina*, *Colletotrichum gloeosporioides*, *Sclerotinia sclerotiorum*, and *Fusarium oxysporum*; the results are shown in Table 1. *Rhizoctonia solani* and *Macrophomina phaseolina* demonstrated pronounced inhibitory effects on growth. These inhibitory effects are more clearly illustrated in Fig. 2, where photos of the cultures are compared with the control assays.

**Table 1:** Inhibitory effect of *Lentinula edodes* crude extract against plant pathogenic fungi

Microorganism	Inhibitory effect (%)
*Rhizoctonia solani*	13.0 ± 0.07
*Macrophomina phaseolina*	5.87 ± 0.03
*Colletotrichum gloeosporioides*	− 1.35 ± 0.16
*Sclerotinia sclerotiorum*	− 8.38 ± 0.16
*Fusarium oxysporum*	− 4.13 ± 0.14

Additionally, *Colletotrichum gloeosporioides*, *Sclerotinia sclerotiorum*, and *Fusarium oxysporum* presented a slower growth cycle; however, an inhibitory action was not observed. In contrast, the fungi *Rhizoctonia solani* and *Macrophomina phaseolina* exhibited better results.

Both fungi, *Rhizoctonia solani* and *Macrophomina phaseolina*, are soil-borne phytopathogenic fungi with a wide host range and geographic distribution, causing significant crop diseases and negatively impacting agriculture and the economy. *Macrophomina phaseolina* is an emerging pathogen that causes diseases like dry root rot and charcoal rot in more than 100 plant families. Depending on the host plant, *Rhizoctonia solani* infection symptoms include seedling damping-off, stem canker, and root or stem rot, and can harm more than 15 families, including maize, soybean, grain crops, horticultural crops, and several other genera (Umer et al. 2023; Ranjan et al. 2024).

β-glucan molecules produced by shiitake may have the ability to inhibit fungal growth. According to the study of Yehia (2022), these polysaccharides showed inhibitory activity against sporulation process in *Aspergillus niger*. This same mechanism of action could have happened with fungi *Rhizoctonia solani* and *Macrophomina phaseolina* in the present work. Rasmy et al. (2010) carried out an in vitro study that reported β-glucan from *Lentinus edodes* presented an antibacterial effect against different kinds of bacteria like *Bacillus megaterium*, *Enterococcus phoeniculicola*, *Klebsiella pneumoniae*, and *Streptococcus* sp. Another study carried out by Hatvani (2001) reported antimicrobial activity of the molecules produced from the submerged fermentation of *Lentinula edodes*. The fermentation broth showed antibacterial activity against *Streptococcus pyogenes*, *Staphylococcus aureus*, and *Bacillus megaterium* (Hatvani 2001).

#### Membrane separation process

Molecules from shiitake are natural and nontoxic to humans; its use could be interesting for different practical applications (Yehia 2022). Therefore, membrane separation was performed with the aim of separating the compounds present in the matrix.

After membrane separation, assays were performed again on the fungi that showed the best results for the antifungal activity analysis. The permeate and retentate fractions were evaluated using membranes with pore sizes of 150 kDa, 20 kDa, and 5 kDa. Table 2 shows the retentate fraction exhibited greater inhibition using the 150-kDa membrane for the fungus *Rhizoctonia solani*. However, the permeate fraction exhibited greater inhibition in the 20-kDa membrane, while the retentate fraction no longer showed an antifungal effect. For the fungus *Macrophomina phaseolina*, membrane separation did not show significant differences for the permeate fractions, but small differences in the inhibitory effect from the separation by pore size of 20 kDa were observed, similar to the results observed for *Rhizoctonia solani*.

**Table 2:** Inhibitory effect against *Rhizoctonia solani* and *Macrophomina phaseolina* of the samples from the membrane separation of the shiitake crude extract

Membrane	Microorganism	Fraction	Inhibitory effect (%)
PES 150 kDa	*Rhizoctonia solani*	Permeate	1.55 ± 0.28^A^
		Retentate	2.07 ± 0.05^a^
	*Macrophomina phaseolina*	Permeate	− 3.8 ± 0.03^B^
		Retentate	− 1.18 ± 0.01^d^
PES 20 kDa	*Rhizoctonia solani*	Permeate	2.61 ± 0.14^A^
		Retentate	− 4.45 ± 0.21^b^
	*Macrophomina phaseolina*	Permeate	0.07 ± 0.61^B^
		Retentate	0.72 ± 0.26^d^
PES 5 kDa	*Rhizoctonia solani*	Permeate	3.13 ± 0.39^A^
		Retentate	− 1.93 ± 0.01^b^
	*Macrophomina phaseolina*	Permeate	3.89 ± 0.56^B^
		Retentate	4.76 ± 0.04^e^

Same letters for same fungus and membrane means no significant difference at 95% (*p* > 0.05) according to Tukey test. Capital letters represent the permeated fraction and lowercase letters represent the retentate fraction

However, these experiments showed lower antifungal activity than the crude extract, indicating that the fractionation did not improve the inhibitory effect under the conditions evaluated. These results suggest that the crude extract may contain multiple bioactive compounds that contribute to the observed antifungal activity, potentially through additive or synergistic effects. Although membrane separation did not enhance the antifungal response, the process remains valuable for the recovery and characterization of shiitake-derived polysaccharides. Therefore, the following sections present the characterization analyses of the obtained fractions to provide a broader understanding of their physicochemical properties.

### FTIR spectrum analysis

**Fig. 3:**
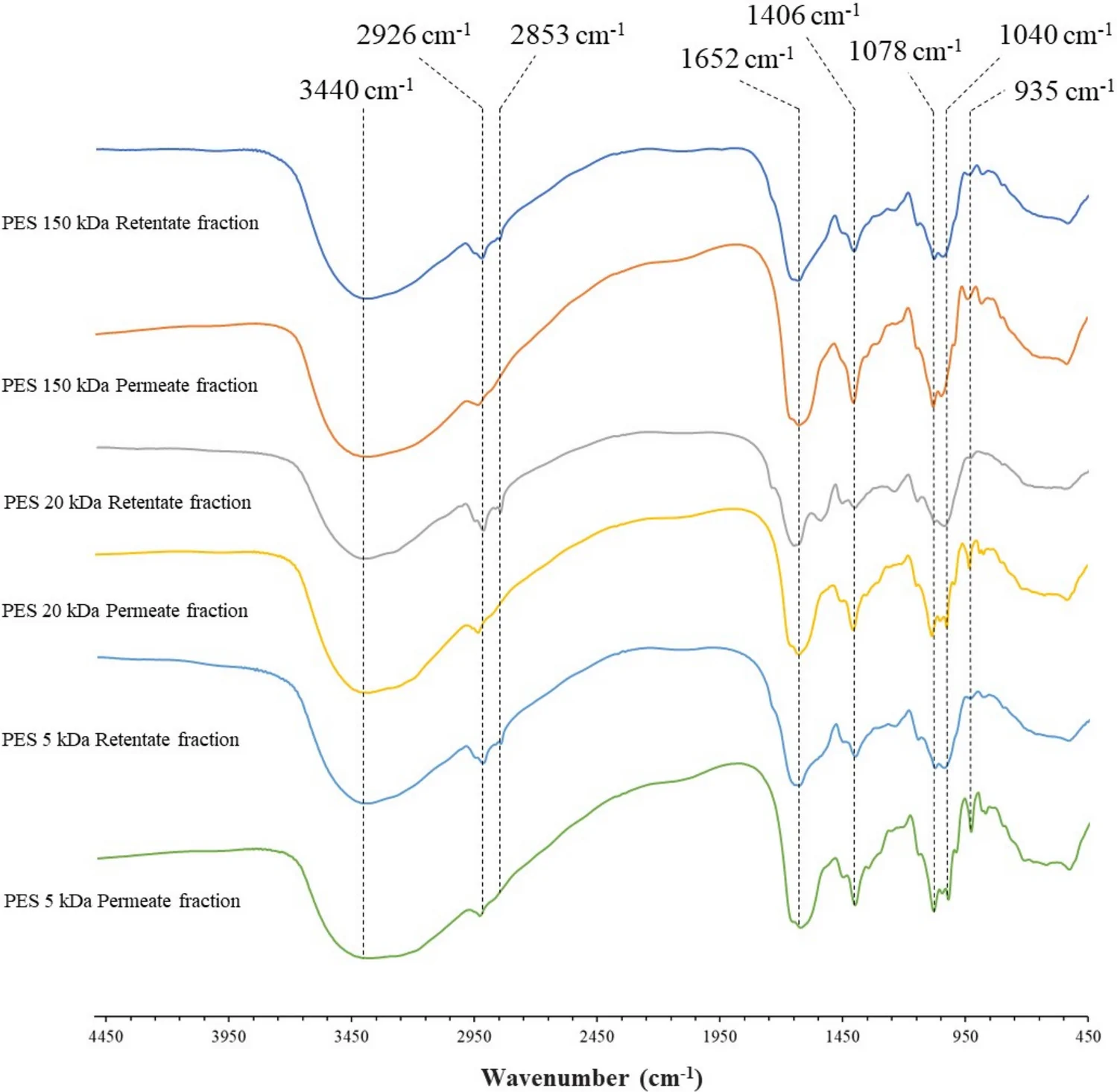
FTIR spectra of the fractions from the membrane separation process

FTIR spectroscopy is commonly utilized to identify and analyze the composition of complex carbohydrate systems (Silverstein and Bassler 1962). FTIR analysis is conducted to elucidate the major functional groups of the polysaccharides from shiitake extract, and the results are presented in Fig. 3. The polysaccharide contained hydroxyl groups, which displayed a peak at 3440 cm^−1^ (range 3700–3100 cm^−1^) (Wang et al. 2019). The bands 2850–2970 cm^−1^ could be attributed to –CH stretching vibration (Vezaro et al. 2022) and the band at 2926 cm^−1^ was present in all samples, whereas the band at 2853 cm^−1^ was noted only in the retentate fractions.

The peak at 1652 cm^−1^ (around 1600–1680 cm^−1^) could be attributed to C=O. The presence of C=O indicated that the polysaccharide contained either amide bonds or carboxylic acid (Wang et al. 2019). The relative absorption peak at 1406 cm^−1^ indicated the characteristic of COO-CH (Vezaro et al. 2022). The peak in the region ranging from 950 to 1200 cm^−1^ suggested the presence of C–O–C and glycosidic bonds in the form of pyranose (Li et al. 2018; Tang et al. 2020). Additionally, the peak at 935 cm^−1^ was characteristic of α-pyranose configuration (Jeff et al. 2013), and was more pronounced in the permeated fractions. The samples presented similar spectra, showing only some differences between the permeate and retentate fractions. The FTIR spectra were consistent with the IR spectra results of polysaccharides from *L. edodes* reported in the literatures.

### Monosaccharides composition and molecular weight of the polysaccharides

The analysis of high-performance liquid chromatography (HPLC) data for monosaccharide content revealed the presence of monosaccharides glucose, rhamnose, and arabinose in all samples. Despite this, the sample’s composition as a mixture of molecules prevents the definitive assertion that all polysaccharides include these simple sugar units in their structures. Polysaccharides consisting exclusively of glucose, known as glucans, are reported by Zhao et al. (2016) which purified polysaccharides with antiproliferative activities from *Lentinus edodes* (Zhao et al. 2016). Furthermore, rhamnose and arabinose from shiitake extract were also identified in the polysaccharides isolated by Li et al. (2018) and Wang et al. (2015) with anticancer and anti-aging capacity, respectively.

A more efficient separation is necessary for a complete understanding of the structures present in each sample. However, based on this work and the data obtained, it is possible to gain a broader understanding that can inform decision-making and assess viability for the next stages of research related to this topic.

**Fig. 4:**
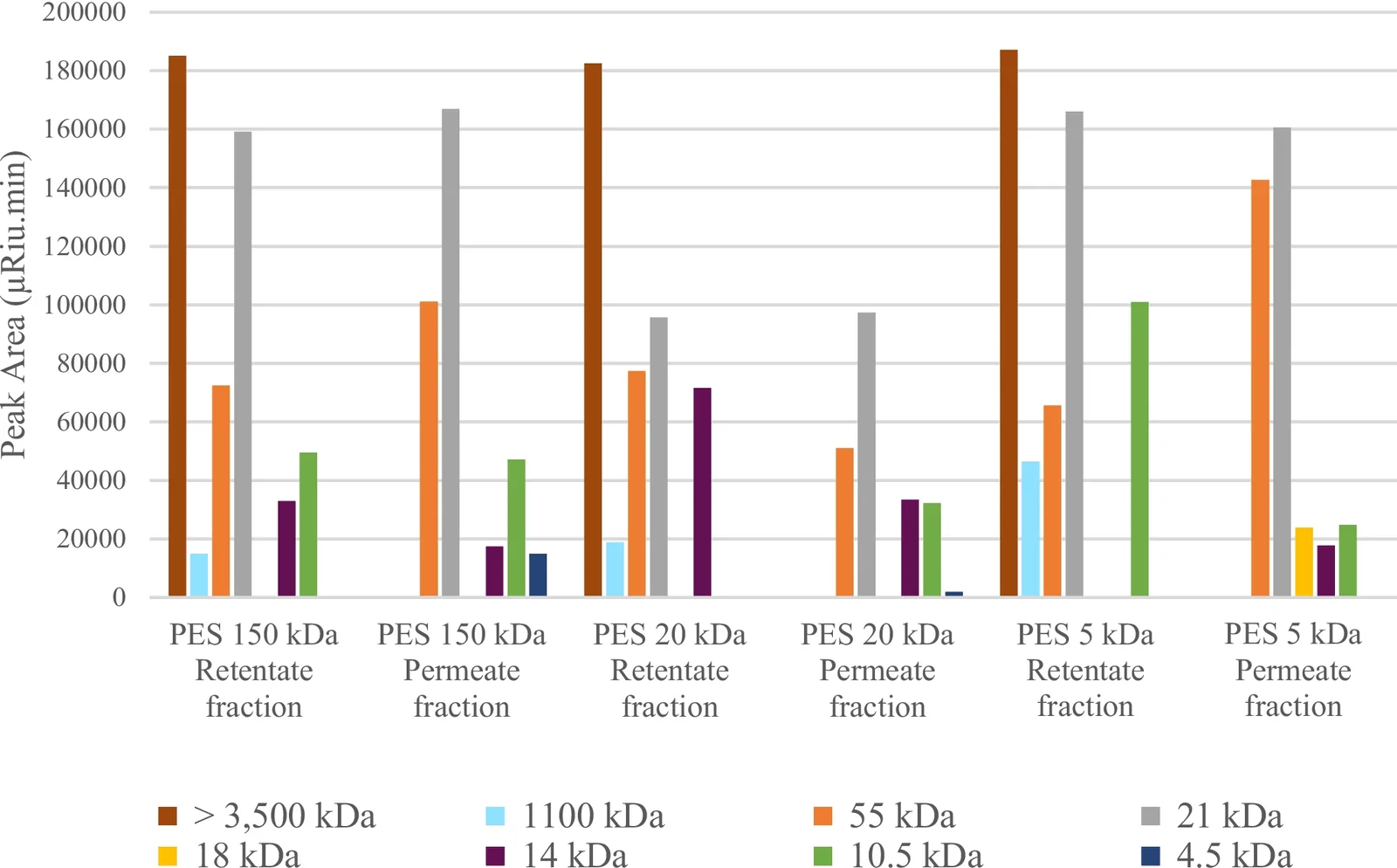
Molecular weight of the polysaccharide content in the permeate and retentate fractions from membrane separation

Figure 4 shows the presence of molecules larger than 3 kDa in both the permeate and retentate fractions after membrane separation. Other separation steps were not utilized due to the aim of the study, focusing solely on assessing the feasibility of the membrane separation process. Consequently, suspended solids and a large number of low molecular weight molecules were observed; however, the permeated fraction did not contain these suspended solids and presented molecules smaller than 55 kDa. This suggests that in processes utilizing ultrafiltration membranes, operations such as clarification, often performed using microfiltration membranes, could potentially be eliminated.

The same groups of molecules were identified in all permeate fractions. Several characteristic factors of the membrane separation process may have been essential for this result, such as the membrane material, the pressure used, and the concentration of solvent in the sample. Additionally, factors like fouling and concentration polarization can influence the separation process (Rosenberg 1995).

Tang et al. (2020) identified three groups of molecules containing polysaccharides obtained by membrane separation processes. They performed microfiltration on the aqueous extract of shiitake to remove insoluble particles, followed by ultrafiltration using membranes of 2.5 kDa, 5 kDa, and 10 kDa. This resulted in fractions with average molecular weights of 14–35 kDa, 14–61 kDa, and ~136 kDa. However, these fractions did not contain purified polysaccharides, and other molecular weight ranges remain to be explored (Tang et al. 2020).

Another study by Morales et al. (2019) achieved fine clarification of the shiitake extract after ethanol precipitation of the polysaccharides. This study utilized a ceramic microfiltration membrane in crossflow operation, and nanofiltration and reverse osmosis membranes for concentration (Morales et al. 2019). These operations produced an extract rich in polysaccharides.

The use of membranes for separation has proven to be suitable for concentrating molecules within a specific molecular weight range. The *L. edodes* extract is rich in polysaccharides, and with adjustments, this method can be efficient and advantageous for practical applications, as the molecules derived from this mushroom have various applications of interest to different industries, such as pharmaceuticals and food. However, to date, no study has successfully purified polysaccharides from shiitake extract using membrane separation alone. Combining this method with other processes, such as ethanol precipitation, microfiltration, dialysis, and using membranes with different materials and pore sizes, may yield purified compounds. Therefore, further studies are needed to confirm the feasibility of this approach.

## Conclusions

This study investigated the recovery, fractionation, and characterization of polysaccharides from expired shiitake mushrooms, and in vitro antifungal activity was evaluated against selected phytopathogenic fungi. The results showed that the crude extract was more efficient than the fractions obtained by membrane separation, with an inhibitory effect of 13.0 ± 0.07% against *Rhizoctonia solani* and 5.87 ± 0.03% against *Macrophomina phaseolina*. For this specific application, the use of the crude extract was sufficient, as the membrane separation process did not improve the inhibitory effect.

Therefore, membrane separation remains relevant for concentrating polysaccharides for other applications. FTIR analysis confirmed the characteristic presence of shiitake polysaccharides, while monosaccharide composition revealed the presence of glucose, rhamnose, and arabinose in all samples. Furthermore, molecular weight analysis indicated that membrane separation is efficient in concentrating and separating molecules within a specific molecular weight range.

The results contribute to the understanding of shiitake-derived polysaccharides and provide a foundation for future studies aimed at assessing their biological activity and practical applicability. Future studies including reference fungicides and additional biological assays may provide further context for the observed antifungal activity. Furthermore, the use of membrane separation demonstrated potential as a sustainable strategy for the recovery and concentration of bioactive compounds from mushroom-derived materials. The recovery of bioactive compounds from mushroom biomass also aligns with ongoing efforts to develop more sustainable and resource-efficient technologies.

## Data Availability

The datasets used and analyzed during the current study are available from the corresponding author upon reasonable request.
